# Optimal Wireless Distributed Sensor Network Design and Ad-Hoc Deployment in a Chemical Emergency Situation

**DOI:** 10.3390/s22072563

**Published:** 2022-03-27

**Authors:** Shai Kendler, Barak Fishbain

**Affiliations:** 1 Department of Environmental, Water and Agricultural Engineering, Faculty of Civil & Environmental Engineering, Technion—Israel Institute of Technology, Haifa 32000, Israel; 2 Environmental Science Division, Israel Institute for Biological Research, Ness Ziona 74100, Israel; fishbain@technion.ac.il

**Keywords:** location-allocation sensor systems, gas detectors, safety management, industrial accidents, air pollution, pareto optimization, genetic algorithms

## Abstract

Industrial activities involve the manipulation of harmful chemicals. As there is no way to guarantee fail-safe operation, the means and response methods must be planned in advance to cope with a chemical disaster. In these situations, first responders assess the situation from the atmospheric conditions, but they have scant data on the source of the contamination, which curtails their response toolbox. Hence, a sensor deployment strategy needs to be formulated in real-time based on the meteorological conditions, sensor attributes, and resources. This work examined the tradeoff between sensor locations and their attributes. The findings show that if the sensor locations are optimal, the number is more important than quality, in that the sensors’ dynamic range is a significant factor when quantifying leaks but is less important if the goal is solely to locate the leak source/s. This methodology can be used for sensor location-allocation under real-life conditions and technological constraints.

## 1. Introduction

### 1.1. Literature Review

Chemical emissions such as toxic gases, vapors, and aerosols from industrial sites threaten human life, health, and the economy. Thus, substantial efforts have been devoted to minimizing the likelihood of these events and mitigating their impact. Chemical detection is essential for a timely and effective response in the case of a disaster. Therefore, considerable work has been devoted to developing leak detection techniques involving indirect and direct detection methods. Indirect methods use surrogate properties such as pressure and temperature changes as indicators of chemical leaks. These indirect sensors provide excellent areal coverage and a rapid response, but they struggle to localize the exact leak location [1]. Sandberg et al. considered the problem of gas leak detection in underground oil pipes [2]. They used a chemical resistor that changed its conductivity in response to the swelling of a conducting layer that resulted from the absorption of organic vapor. The detector consisted of a 2 km long cable placed close to the pipeline. The response time to leaks was ~15 min with a spatial resolution of 20 m.

This type of sensor furnishes a solution to a critical challenge in the sensor location-location problem: providing extensive areal coverage with limited resources. However, indirect sensors may not be specific enough and depend on prior knowledge of the location of potential leak sources. Chemical detectors can complement the indirect monitoring of leaking materials. For example, Guo et al. developed an early-warning gas leakage monitoring system based on a Wireless Distributed Sensor Network (WDSN) [3]. WDSNs provide a fast response (seconds) and good areal coverage thanks to their low price, high portability, and communication capabilities. Similarly, Somov et al. developed sensors that can be deployed as WDSN within an industrial site [4]. However, operating WDSN in harsh environments reduces performance over time [5]. Hence, sensors must be protected [6,7] and undergo periodic maintenance [4].

Furthermore, when deploying WDSN in a potential chemical disaster area, the sensors must be located to capture the pollution dispersion optimally. This optimization dilemma is known as the location-allocation problem [8,9]. Legg et al. described the optimal placement of chemical detectors inside a chemical plant which minimizes the time needed to sound the alarm in various scenarios [10]. Ndam et al. used multiobjective optimization for the general case of sensor deployment [11]. They searched for a solution that provides maximal source coverage with a minimum number of sensors, assuming that each sensor covers an area with a specific radius. Lepley et al. developed an entire system for first responders in the case of toxic chemical release. Their system is comprised of several gas detectors, meteorological communication modules, and an optimization unit for sensor deployment that accounts in real-time for changes in meteorology and gas plume properties [12]. Marjovi and Marques developed a mobile sensor device mounted on a swarm for odor detection using Powell’s conjugate gradient descent method to maximize the detection probability [13]. These mobile systems, when combined with low-cost sensors, are gaining popularity since they provide extensive direct sensing areal coverage as well as adaptability to evolving situations [14], which is advantageous for many cases related to emergency situations [15].

### 1.2. Motivation and Innovation

The above body of work highlights the central issue in a catastrophic event involving a gas leak: the need for chemical sensing in a large area using stationary or mobile sensors. This type of operation requires considering numerous parameters related to sensor properties, maintenance, placement, and resources. Previous studies solved the location-allocation problem by assuming sensors with known and fixed attributes, for example, [12] and [16]. Some theoretical studies assumed the sensors’ attributes exceed the application requirements (ideal sensor), for example [17]. No previous work has combined the problems of sensor design with the sensor location-allocation problem into one holistic approach. Solving these two problems together constitutes a powerful approach to evaluating the mutual effects of sensor attributes and location and provides guidelines to make informed decisions as to sensor design and application.

This combined algorithm for sensor location-allocation and design is oriented toward optimal responses in chemical emergencies. The tradeoffs between sensor attributes (sensitivity, dynamic range, and the number of detectors) and their location are examined for the case of first responders called upon to monitor chemical leaks outside a chemical plant. The scenario is cast as a multiobjective optimization problem, thus making it possible to incorporate different environmental factors and detector attributes into the model. This approach leads to:

1. Defining optimal sensor locations.
2. Identifying a match between sensor attributes and the emergency problem at hand.
3. Estimating the possible errors deriving from the sensor attributes.

## 2. Methodology

### 2.1. Problem Formulation

Let Ω be the region of interest which in our case is an industrial site, {*S*} is a set of possible leak sources, where each source, *s* ∈ {*S*} is located in $\omega_{s}\in \Omega$ and emits $q_{s}\left(t\right)$ [kg/s]. Similarly, the set {R} is the set of sensors, where each sensor, r ∈ {R}, is located in $\omega_{r}\in \Omega$ and records a pollution level of $c_{r}\left(t\right)\;\left[\frac{\mu g}{m^{3}}\right]$. The list of potential leak locations is known in advance and is derived from the infrastructure configuration. Here, each such location is considered a potential source that can either be active if $q_{s}\left(t\right)>0$ or inactive. Without loss of generality, if the potential source locations cannot be deduced or limited, then Ω can be divided into a set of catchments, {*ω*} ∈ Ω, where each catchment can host an active source. In a real-life scenario, the active ($q_{s}\left(t\right)>0$) source locations, $\left\{\omega_{s}\right\}$ and emission rates, $\left\{q_{s}\right\}$ for all *s* ∈ {*S*} are *unknown*, whereas the sensor locations, $\left\{\omega_{r}\left(t\right)\right\}$ and recorded values, $\left\{c_{r}\left(t\right)\right\}$, for all *r* ∈ {*R*} are *known*.

The expected gas concentrations are calculated using a dispersion model. To do so, let $m_{sr}$ be the pollution transfer function of the dispersion model, which associates sensor r’s readings, $c_{r}\left(t\right)$, with the emissions of source *s* [18]. Generalizing this notion, the model’s estimation of the pollution level in *ω* ∈ Ω, due to $q_{s}\left(t\right)$ is given by:

(1) $$\left.c_{\omega }\left(t\right)=m_{s\omega }\cdot \;q_{s}\left(t\right)\right|\;\forall \omega \in \Omega ,\;\forall s\in \left\{S\right\}\;$$

Ideally, a detector should have a minimum detectable level (MDL) of 0 kg/m^3^, and an infinitely wide dynamic range (DR) in which the sensor response to the chemical is linear, i.e., a Saturation Level (SL) that goes to infinity. For non-ideal detectors and a pollution level of $c_{r}\left(t\right)$ on the test site, the sensor response function is given by:

(2) $$\hat{c_{r}}\left(t\right)=\left\{\begin{array}{c} \begin{array}{c} \begin{array}{cc} \;\;\;\;\;\;\;\;0, & \;\;\;\;\;\;\;\;\;\;\;\mathrm{MDL}>c_{r}\left(t\right) \end{array}\\ \begin{array}{cc} c_{r}\left(t\right), & \mathrm{MDL}\leq c_{r}\left(t\right)\leq \mathrm{SL} \end{array} \end{array}\\ \begin{array}{cc} \;\;\;\;\;SL, & \;\;\;\;\;\;\;\;\;\;\;\;\;\;\;\;\mathrm{SL}<c_{r}\left(t\right) \end{array} \end{array}\right.$$

Based on the notation above, the optimization problem can be formulated such that the measurement error, ${\hat{c}}_{r}\left(t\right)-c_{r}\left(t\right)$, is as small as possible at the measurement points:

(3) ΨErr=∑r∈Rc^rt−crtcrt

This cost function assumes that increasing the detectors’ DR, and decreasing their minimum detectable level, MDL, increases their price [19,20], and operational cost. If the network consists of a homogenous set of sensors and the installation cost is fixed for all *ω* ∈ Ω, Equation (4) becomes:

(4) $$\Psi_{Cost}=\left|R\right|\cdot \left(\frac{1}{{\mathrm{MDL}}_{r}}+{\mathrm{DR}}_{r}+\mathrm{SC}\right)$$

In our scenario, all the sensors were placed outside the industrial site, resulting in a uniform installation cost.

As outlined in (5), the cost function enables continuous changes in the detectors’ attributes, making it possible to explore the tradeoffs between cost and performance. The cost function can be modified for each specific problem. For example, if sensors are chosen from a specific inventory with known prices and attributes, the cost function can be reformulated accordingly [16].

The resulting multiobjective function is then:

(5) $$F=\left[\begin{array}{c} \Psi_{Err}\\ \Psi_{Cost} \end{array}\right]\;$$

The two objectives in *F* conflict since decreasing the detectors’ array cost will also increase the measurement error and vice-versa. Thus, the goal is to find the set of locations in space, i.e., the set $\left\{\omega_{r}\right\}\in \Omega$ and the sensors’ attributes, DR and MDL, that will minimize both the cost and measurement error. The tradeoffs between $\Psi_{Err}$ and the source estimation discrepancies are discussed below.

### 2.2. Test Site

Computation, in terms of the scenarios described below, requires knowing $c_{\omega }\left(t\right)$, which can be obtained either in a controlled experiment or by using a gas dispersion model in a simulated arena. The Graz Lagrangian Model-GRAL [21,22,23] was used to generate this information in this work. This model was developed to assess air pollution in complex terrains. For example, Rafiei and Sturm used GRAL to study the dispersion of carbon monoxide around urban tunnel portals [21]. Berchet et al. used GRAL to compute NOX concentrations in Zurich compared to measured data [22]. They showed that GRAL generated a reasonably accurate estimate of the NOX concentration. Öettl implemented a refined microscale flow field model in GRAL [23]. Their flow field model was based on the Reynolds-averaged Navier–Stokes equations [24,25] and used a turbulence model to account for obstacles in the flow field.

**Figure 1:**
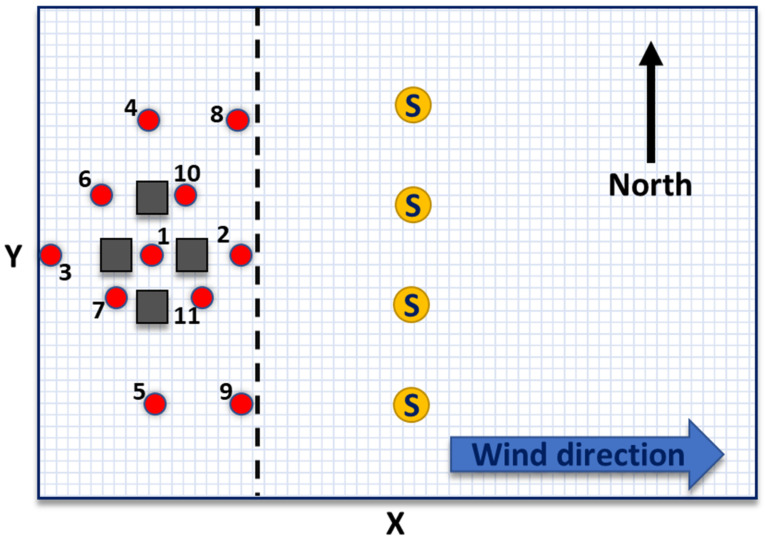
Diagram (not to scale) of the setup of the test site: a flat 1225 m × 850 m surface is partitioned into 25 m × 50 m grid units marked by the gray lines. Four (50 m × 50 m × 20 m) buildings are located in the western section of the site and are marked by squares. Possible leak sources are indicated by the red dots. The yellow circles depict an example of a possible sensor (S) array positioned perpendicular to the wind direction. The dashed blue line 375 m from the western side represents the industrial site fence.

Here, the test site was a flat (1225 m × 850 m) area with eleven possible leaks and four (50 m wide, 50 m long, 20 m high) buildings. The gas concentration was computed in 833 receptors evenly spread 3 m above ground level, creating a 25 m × 50 m grid. The test site is depicted in Figure 1, and the computational specifics of the gas dispersion model are listed in Table A1 in Appendix A.

### 2.3. Solving the Optimization Problem

The optimization problem above is NP-hard (by reduction to the knapsack problem [16]). To solve this type of problem, a heuristic approach is required. Here, BORG, a Multiobjective Evolutionary Algorithm (MOEA), was utilized. BORG is a hyper-heuristic global optimization framework that uses genetic algorithms to search for an optimal set of decision variables, i.e., the number of sensors, MDL, DR, the distance from the fence, the leak location, and the rate [26,27,28]. The Borg MOEA initiates the search for the optimal set by random generation of its initial population of candidate solutions. Then the algorithm projects the decision variables on F based on the two resulting objectives ($\Psi_{\mathrm{Err}}$ and $\Psi_{\cos t}$). The Borg MOEA rewards the sets of decision variables that dominate the competing alternatives (i.e., are better on both objectives). The decision variables are then modified to determine whether a better solution can be generated. This process is repeated until a high-quality approximation of the Pareto frontier is attained. Using a stochastic restart mechanism, the BORG MOEA avoids premature convergence to a local optimum and thus can output a diverse set of solutions.

**Figure 2:**
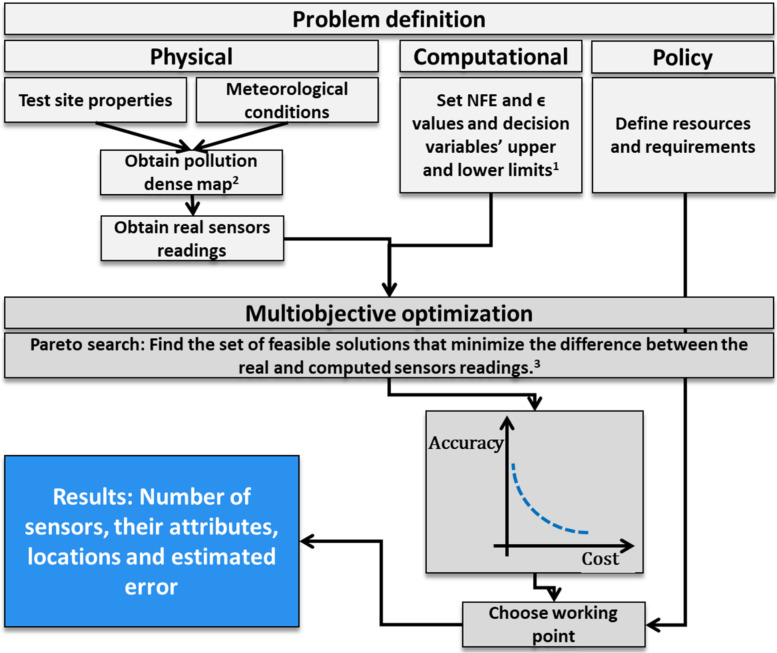
Flowchart of the method. 1. The decision variables are listed in Table 1 below. 2. Here, the pollution-dense map was obtained using the Lagrangian atmospheric dispersion model. 3. During the optimization, the sensors’ readings are computed using the same model based on assumed leak rates and sensor attributes (decision variables). If the assumed leak rate is close to the actual one and the sensor locations and attributes are optimal, the difference between the computed and the real sensors’ reading will be minimal.

The BORG’s search results are affected by the required level of precision of each condition; in this case, $\varepsilon_{Err}$ and $\varepsilon_{Cost}$ (epsilon) control the minimum resolution or spacing of the solutions. As the epsilon values decrease, a more fine-grained set of solutions is obtained. Another factor that affects the solution is the Number of objective Function Evaluations (NFE). Once the function has been evaluated NFE times, the search is stopped, and the best solution obtained is taken as optimal. The relative effect of these parameters on the solution can be quantified by setting a specific problem and solving it with different epsilons and NFE values. Here, sixteen NFE values varying from $1\times 10^{3}$ to $1\times 10^{5}$, twelve values of $\varepsilon_{Cost}$ varying from 1 to $8\times 10^{-4}$ and sixteen values of $\varepsilon_{Err}$, from 10 to $2\times 10^{-4}$ were used to create 3072 such run-time parameter combinations for the case where the leak rate and location were known. The results showed that the factor that affected the error the most was NFE. For NFE > $3\times 10^{4}$ the difference between the set and actual leak rate was negligible and stable to changes in $\varepsilon_{Err}$ and $\varepsilon_{Cost}$. Based on this analysis, the run-time parameters in this work were set to $\varepsilon_{Err}=0.01$, $\varepsilon_{Cost}=0.1$, and NFE = 10^5^. A flowchart of the method is presented in Figure 2.

The MOEA algorithm has stochastic elements that can result in different outcomes for the same problem, even when the same run-time parameters are used. Hence, each leakage configuration problem was solved 50–500 times, using the same run-time parameters, to verify that the results converged consistently. Once the optimization problem has been solved, the sensors’ computed locations and attributes and leak properties (location and rate) can be obtained. The computed sensor array’s performance is evaluated by comparing the actual leak properties to the computed properties.

## 3. Results and Discussion

### 3.1. High-End Sensor Array

Performance was tested on a high-end detector array. The lower and upper bounds of the decision variables (the number of detectors, their MDL and DR) were set to enable the BORG to achieve high accuracy by using a large set of detectors with a large DR and very low MDL. Table 1 lists the lower and upper bounds of the decision variables for this high-end detector set.

**Figure 3:**
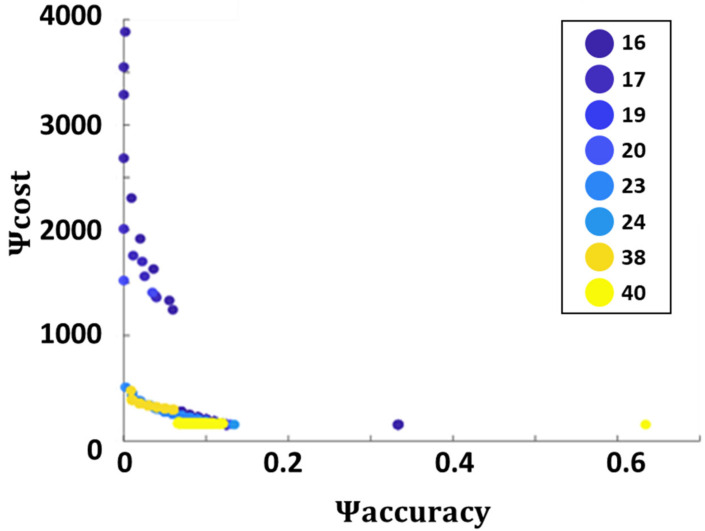
The Pareto frontier was obtained by using a high-end sensor array when source #5 was leaking at a rate of 2.5 kg/s. Feasible solutions obtained at different detector array distances are marked with solid circles. Note that the feasible accurate solutions are located at 38 and 40 gu from the fence.

**Figure 4:**
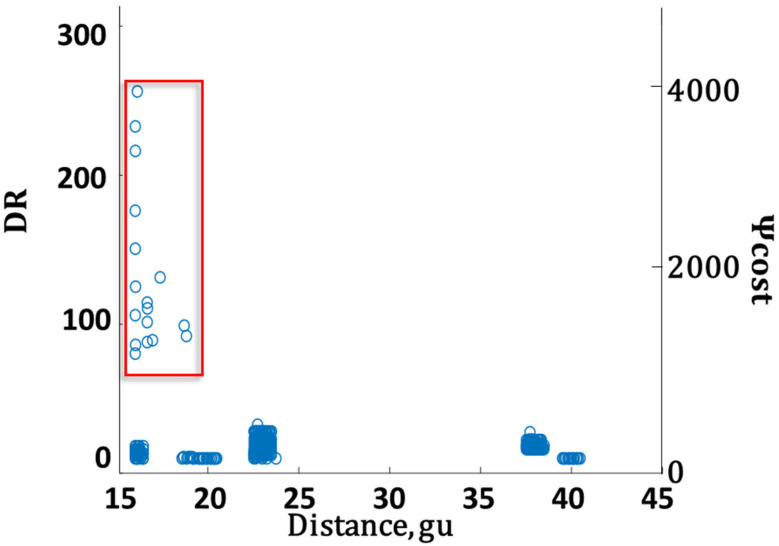
The sensors’ DR (left axis) and cost (right axis) distribution for different distances for all feasible solutions obtained when using a high-end sensor array. The red rectangle frames the exceptionally accurate feasible solutions that result from using detectors with a very high DR.

**Figure A1:**
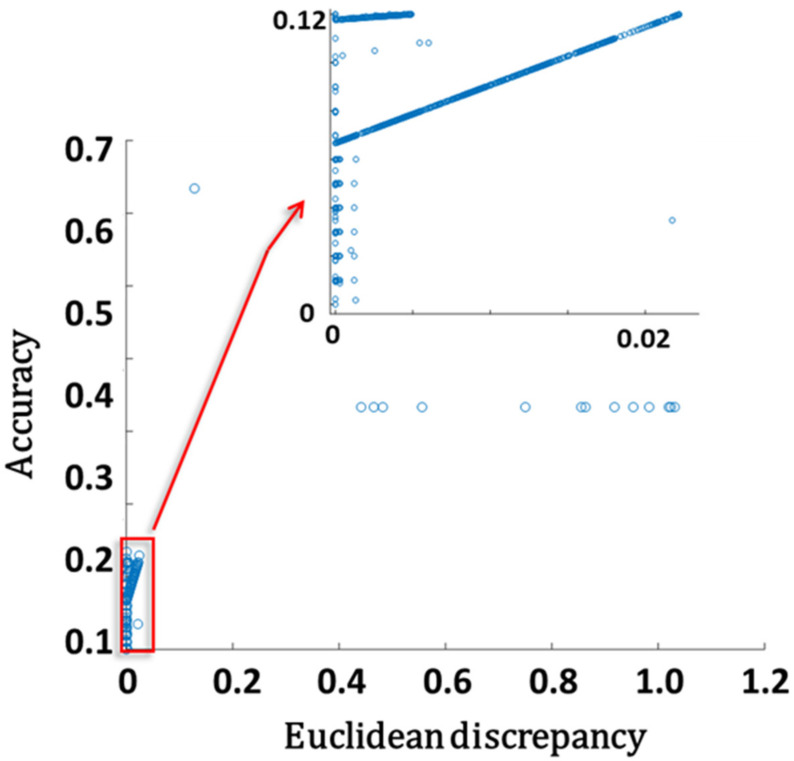
A Pareto frontier computed for a 2.5 kg/s leak from source #5. Different colors mark the feasible solutions obtained at different detector array distances. The graph shows a zoom-in on the $\Psi_{accuracy}\leq 0.01$ range of the Pareto frontier.

Figure 3 shows an example of the Pareto frontier obtained when source #5 was leaking at a rate of 2.5 kg/s. This computation was repeated 500 times. Figure 3 presents feasible solutions from all of these runs. Feasible solutions obtained at different distances are marked in different colors. Note that the distances are given in grid units (gu); to convert to actual distances, multiply by 50 m/gu. As expected, there was a tradeoff between accuracy and cost. Achieving more than 1% accuracy came at the cost of a relatively low sensor at distances as high as 38 gu. Zooming in to the high accuracy region of the graph shows that decreasing the distance increased both accuracy and cost by a factor ~3–6 (see Figure A1 in Appendix B). The tradeoff between the sensor array distance and cost was also examined. Figure 4 presents the detector array cost and DR distributions at different distances for all feasible solutions. It shows that deploying the detector array close to the fence in some cases resulted in a steep rise in cost. However, these expensive configurations also provided higher accuracies. The increase stemmed from the fact that when nearer the source, the variations in the gas concentration were higher than at distances farther away, where the gas plume had expanded.

Solving the optimization problem resulted in minimizing $\Psi_{Err}$, which raises the question of the relationship between $\Psi_{Err}$, and the deviation from actual measurements. This deviation was thus estimated for each feasible solution using Euclidian metrics:

(6) $$Euclidean\;discrepancy=\sum_{s}\sqrt{{\left(q{\left(t\right)}_{s,actual}-q_{s,computed}\right)}^{2}}$$

where $q_{s,\;actual}$ and $q_{s,\;computed}$ were the actual and computed leak rates from source s.

**Figure A2:**
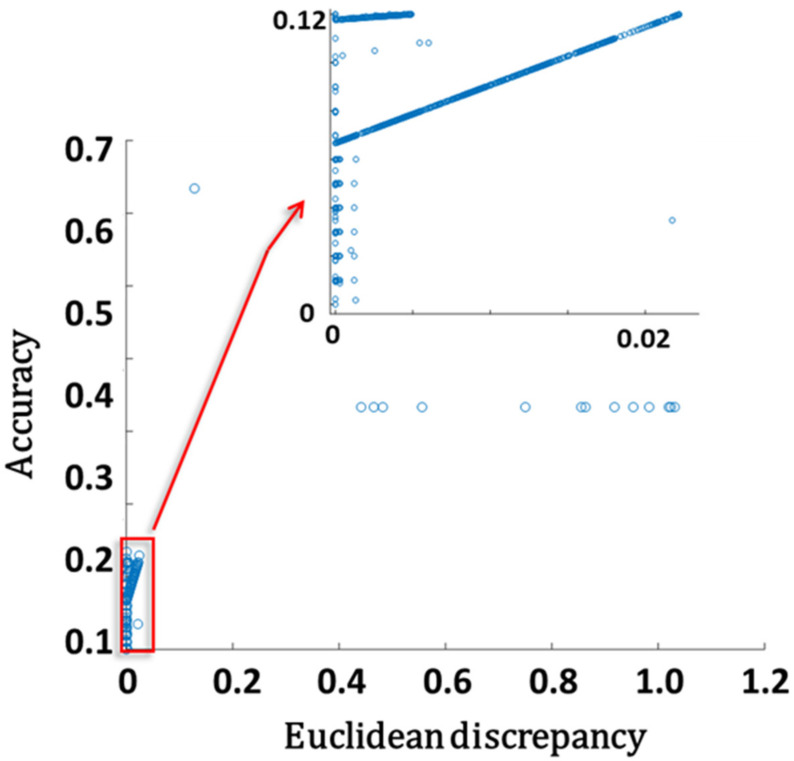
The computed accuracy ($\Psi_{Err}$) vs. the Euclidian discrepancy using a high-end detector array when source #5 is leaking at a rate of 2.5 kg/s. The upper part shows an enlargement of the accurate solution region as marked by the red rectangle.

A Euclidian discrepancy close to zero indicates that the computed solution was close to the actual situation for both the leak location and rate, suggesting that this solution was reliable. Comparing $\Psi_{Err}$ to the Euclidian discrepancy showed that a low value of $\Psi_{Err}$ also resulted in a low Euclidian discrepancy; thus, the computed result was more reliable (see Figure A2 in Appendix B). This relationship between the computed value ($\Psi_{Err}$) and the Euclidian discrepancy makes this algorithm a helpful metric as in many cases, the leak properties are unknown, whereas $\Psi_{Err}$ can be computed using experimental measurements of the actual gas concentration.

### 3.2. Practical Sensor Array

**Figure 5:**
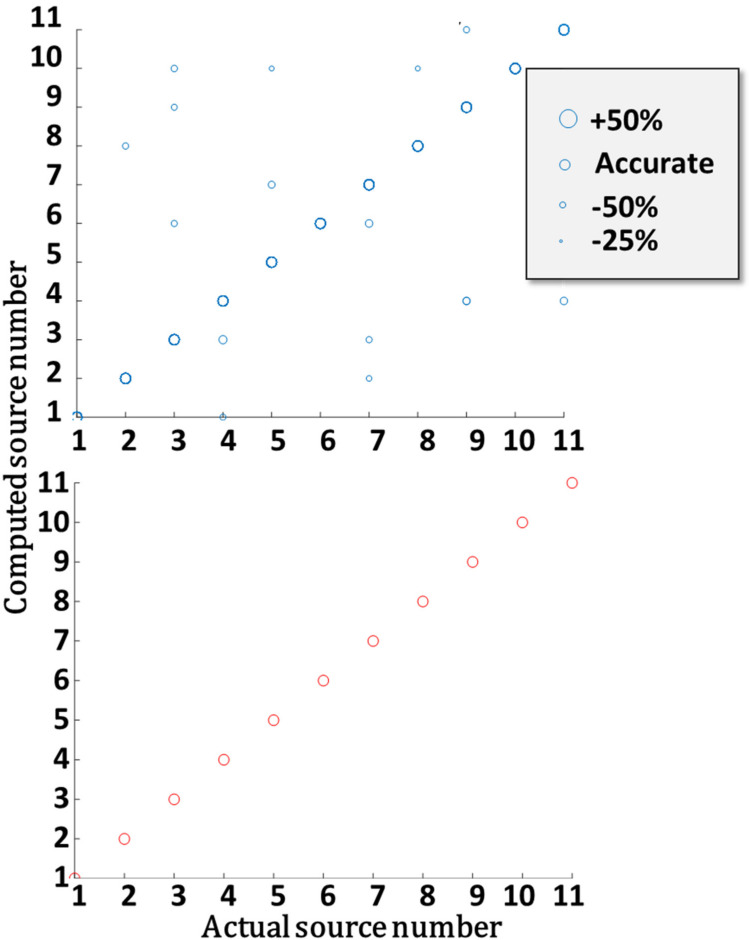
Computed vs. actual leak sources using a practical detector array. (**Top**): results using all feasible solutions. Computed leak rate accuracy is presented graphically, where underestimation of the leak rate is depicted in small circles and overestimation in large circles; see box legend. (**Bottom**): Same as above, only for the most accurate feasible solutions (T5) for each situation.

For this detector array, the MDL and DR were set to 3 kg/m^3^ and 10, respectively. Computations were performed with 16–17 detectors placed at a distance of 20 to 44 grid units from the industrial site fence. The performance of this array was tested for all possible cases in which one source was leaking (at a leak rate of 2.5 kg/s). For each case, the computation was repeated 50 times. The upper part of Figure 4 shows a scatterplot of the computed source locations (1–11) as compared to the actual one for all feasible solutions. The circle size indicates the discrepancy between the computed and actual leak rates. Solutions for which the leak rate was underestimated are marked with smaller circles and overestimation with larger circles. About 97% of the feasible solutions accurately estimated the leak source locations and rate; hence, the symbols in the figure overlap. These solutions fall on the diagonal of the figure. However, some feasible solutions wrongly identified the leak source and rate. The bottom part of Figure 5 shows that focusing on the 5% most accurate feasible solutions (T5) resulted in a reliable leak rate and location determination. Note that selecting the T5 solutions required choosing the solution having the lowest computed $\Psi_{Err}$ values but did not require any prior information on the problem, which indeed may be unavailable in actual cases.

### 3.3. Resiliency to Changes in Problem Conditions

#### 3.3.1. The Effect of a Reduction in the Number of Detectors and Their Attributes

Here, the above computation was repeated using different numbers of sensors for a practical detector array (MDL = 3 kg/m^3^, DR = 10). When reducing the number of sensors from 17 to 6, there were fewer accurate solutions. Even under these unfavorable conditions, all T5 solutions were reliable, and the estimations of all 11 possible sources were accurate. A further drop in the number of detectors to four resulted in decreased performance, and when considering the T5 solutions, one out of 11 sources was wrongly identified. Using an array of only two detectors resulted in an unacceptable error rate, where about ~80% of the feasible solutions were unreliable even when only using the T5 solutions.

Improving the detector array performance (MDL = 1.5 kg/m^3^, DR = 30) provided accurate results even with four detectors; for the T5 solutions, all 11 sources were located and estimated accurately. However, even with this improved detector array, reducing the number of detectors to two resulted in unreliable results, and only three out of the 11 sources were identified correctly.

To test the limits of the algorithm, a detector array with exceptional detection capabilities (MDL = 0.001 kg/m^3^, DR = 500) was tested. Even with these exceptional detectors, the results were inaccurate when the detector array consisted of two detectors. This result clarifies that increasing the detector performance does not compensate for the decrease in the number of sensing points.

#### 3.3.2. Varying Leak Rates

**Figure 6:**
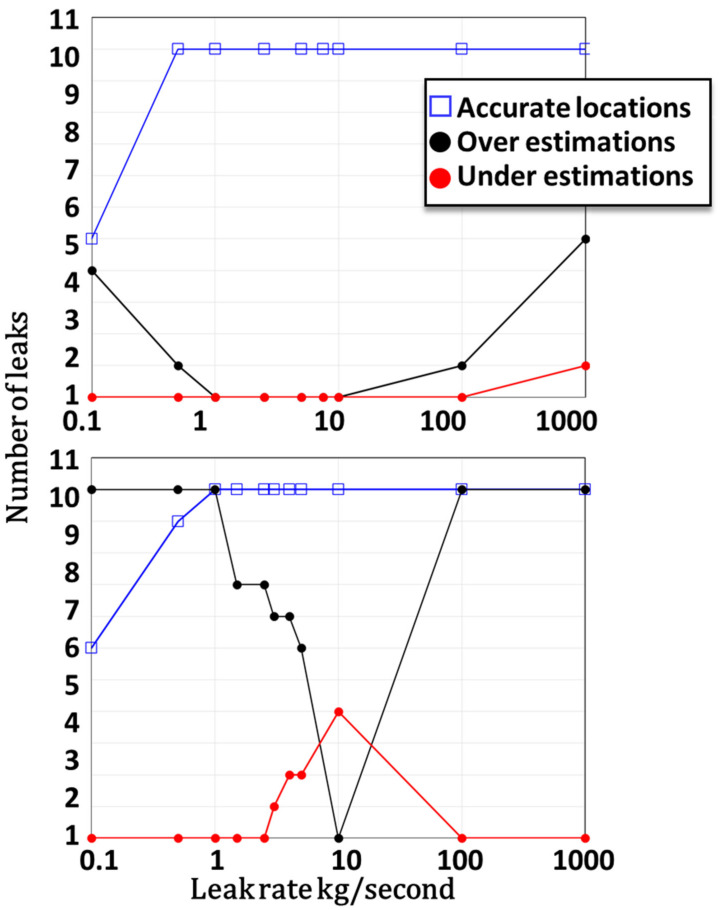
(**Top**): number of accurate leak locations (blue squares), number of overestimated leaks (black dots), and number of underestimated leaks (red dots) as a function of the leak rate for a six-detector array with an MDL of 3 kg/m^3^ and a DR of 30. (**Bottom**): same for a six-detector array with an MDL of 3 kg/m^3^ and a DR of 1 (binary detector array).

The above computations were performed at a constant leak rate of 2.5 kg/s. Since the detector array attributes are set before any event occurs, it is crucial to verify that the detector array is effective for a wide range of leak rates. The top part of Figure 6 shows the leak rate effect on the reliability of the solutions when using an array with six detectors with an MDL of 3 kg/m^3^ and a DR of 30. At low leak rates, the estimated leak locations and rates were inaccurate. For example, at a leak rate of 0.1 kg/s, only five out of the 11 leak sources were accurately located, such that the typical error of the estimated leak rate, in this case, was over 50%. At leak rates from 0.5 to 10 kg/s, both the quantification and location were accurate (quantification range). At higher leak rates (up to 1000 kg/s), the location of leaks was accurate, but the leak rate was inaccurate due to saturation of the detectors; this range of emission rates is referred to as the detection range.

The ability to accurately estimate a leak rate depends on the detectors’ DR. As the DR decreases, so does the quantification range. The lowest possible DR corresponds to a binary detector that provides zero readings when the gas concentration is lower than the MDL, and a constant reading, SL, once the gas concentration exceeds this threshold. The bottom part of Figure 6 shows the number of accurately located leak sources and the number of over- and under-estimated leak rates when using a six-detector array with an MDL of 3 kg/m^3^ and a DR of 1 (binary detector array). Quantification emerged as impractical with a detector array containing only binary detectors. However, the detection range was hardly affected at all, and the computed leak locations were accurate even in this extreme configuration.

The computations presented here suggest that a binary sensor array can be used if the main goal is to determine the leak location. This finding is significant as operating detectors in extreme field conditions is a highly demanding task. Hence, procuring detectors at a lower cost or setting a more lenient maintenance routine are factors that a decision-maker must consider. Admittedly, most detectors have monitoring capabilities (DR > 1), and binary chemical detectors are rare. Calibration and validation are required to take full advantage of these monitoring capabilities. Alternatively, the detectors can be treated as though they were binary, i.e., by considering any reading below a certain threshold as zero and one once the detector reading exceeds this value. This helps avoid meticulous calibrations, thus significantly reducing operational costs.

**Figure A3:**
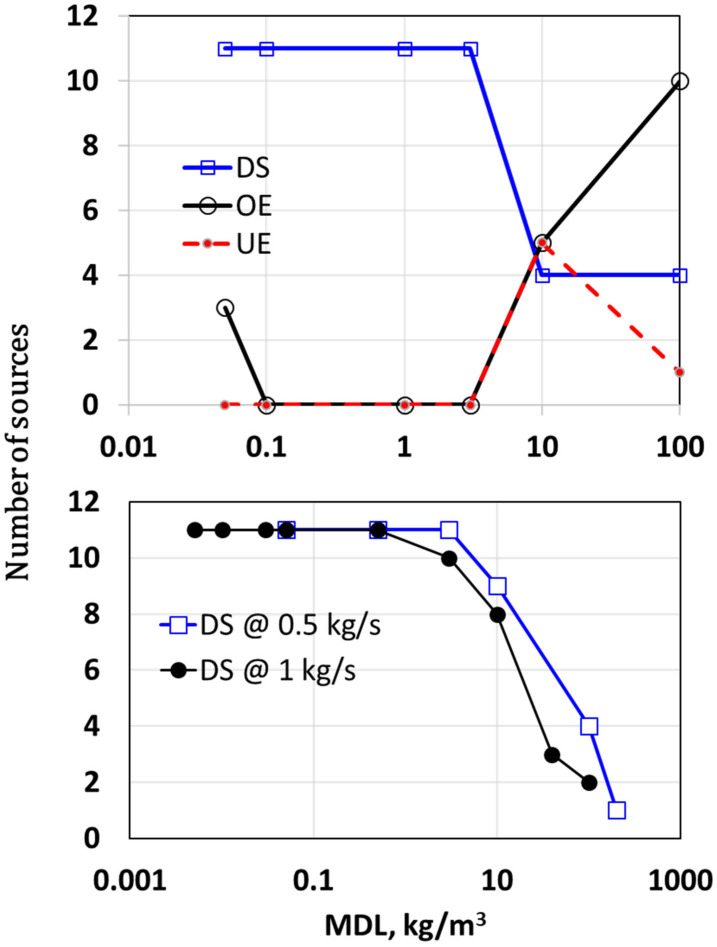
(**Top**): number of accurate leak locations (DS—blue squares), number of overestimated leaks (OE—black circles), and number of underestimated leaks (UE—red dots) as a function of MDL for the six detector array with a DR of 30 at a constant leak rate of 0.5 kg/s. (**Bottom**): number of accurate leak locations (DS) as a function of MDL for the six detector array with a DR of 1 at a constant leak rate of 0.5 kg/s (blue squares) and 1 kg/s (black circles).

Similarly, the effect of the MDL on method performance was explored for both a binary sensor array and sensors with DR = 30 for a constant leak rate. When DR = 30, the MDL could vary by almost two orders of magnitude without compromising the detection range, whereas the quantification range was slightly smaller than the detection range. A similar detection range was found for the binary detectors (see Figure A3 in Appendix B).

## 4. Conclusions

The industrial manipulation of chemicals involves the risk of chemical leaks. In case of an accident, first responders arrive on the scene and take steps to mitigate the situation to reduce the risk to the population and eventually stop the chemical leak. One of the primary tasks is to locate the leak and, if possible, assess its rate. Since the leak location is unknown, deploying sensors can be complicated, and the number of sensors must be increased to accommodate all possible situations [17]. WDSNs are fast, portable, and have excellent communication capabilities, thus making them good candidates for such applications [3].

Furthermore, WDSN can be mounted on a mobile platform which provides good areal coverage and excellent adaptability to evolving situations. These modes of operation are possible given the size and weight of WDSN and their lower cost compared to state-of-the-art air monitoring equipment. However, despite their excellent properties, the procurement and operation of WDSN in industrial environments are still challenging [4]. Hence, the sensors’ location-allocation must be addressed even for these sensors.

Previous works have described various approaches to solving the location-allocation problem of chemical sensors. Each specific application requires different sensors and sensing strategies; hence several methods have been put forward. For example, Legg et al. aimed for a solution that minimizes the response time to a chemical leak in a chemical plant [10]. Ndam et al. described a sensor deployment that maximized the area monitored by a minimal number of sensors [11]. Lepley et al. developed a system that includes various chemical and meteorological sensors for first responders [12]. Marjovi and Marques developed a mobile sensor device mounted on a swarm design to maximize the probability of detecting odors [13]. Lerner et al. used an array of sensors chosen from a specific inventory with fixed attributes to monitor air pollution in large areas. Aharoni et al. suggested an alternative approach for solving the location-allocation problem in close chambers. They managed to map gas concentration using a single detector by simultaneously collecting samples from several points at predetermined mixing ratios. The gas concentration at each sampling point is computed using a dedicated algorithm that uses the sensor readings and the mixing ratios [29]. These key studies used sensors with fixed attributes and aimed to solve the location-allocation problem under the constraint of a specific sensor array. By contrast, the current study evaluated the tradeoff between cost and performance of the sensor array. Cost is a function of the number of sensors and their attributes, whereas performance relates to the possibility of locating the leak and determining the emission rate using sensing information. This method provides a systematic procedure for choosing the number of detectors, their attributes (minimum detectable level, dynamic range), and locations.

It is based on a Lagrangian gas dispersion model for computing the gas concentration in the air caused by leaks from various possible locations and rates in the test site. A multiobjective approach can then be used to find the tradeoff between the accuracy and cost of the sensing network using the Borg MOEA framework. The findings showed that accurate, feasible solutions are also reliable ones. This method thus constitutes a valuable tool for the decision-maker when considering sensor arrays to detect chemical leaks. Specifically, as the requirements for accurate estimation of the leak rate increase, the number of detectors should increase, and their attributes should be enhanced. If there is a need to operate at lower costs, effective determination of the leak location is possible with binary detectors that indicate values above or below a given threshold. Similarly, this method provides insights into the MDL needed to detect leaks at different rates typical to each site.

This study is part of a larger ongoing project to provide means and methods for effective gas monitoring for first responders and regulators. Here, it was tested on a simulated dataset. The next step will be to challenge this method with real-life data and detectors.

## Figures and Tables

**Table 1:** Upper and lower bounds of the decision variables.

Decision Variable	Lower Bound	Upper Bound	Units
Leak rate	0	10	kg/s
Number of detectors	16	17	
Detector array distance	15	45	Grid units (1 gu = 50 m)
MDL	0.1	3	kg/m^3^
DR	10	300	

## Data Availability

Not applicable.

## Appendix A. The Graz Lagrangian Model—GRAL

The gas concentration was estimated under various conditions and constituted crucial input for the work presented here. This was achieved by using an atmospheric dispersion model in a simulated arena in known meteorological conditions. In this work, we used a Lagrangian model implemented in the “Graz Lagrangian Model—GRAL”. This model was developed to assess air pollution in complex terrains as a collaboration between the Graz University of Technology and the Department of Air Quality, Government of Styria. Table A1 below lists the parameters used in this work.

**Table A1:** List of parameters used for the GRAL computation.

	Parameter	Value	Units
Operational parameters	Operation duration	3600	s
	Dispersion time	1200	s
	Particles per second	200	1/s
Source Parameters	Emission rate	10	kg/s
	Source diameter	0.6	m
	Exit temperature	353	K
	Emitted gas	CO	
	Deposition	No	
	Exit velocity	6	m/s
	Height	10	m
Meteorology	Wind direction	West	
	Wind velocity	2	m/s
	Atmospheric stability class	C	
	Surface roughness	0.2	m
	Roughness of building walls	0.01	m
	Horizontal grid resolution	5	m
	Vertical thickness of the first layer	2	m
	Vertical stretching factor	1.01	
	Number of cells in the z-direction	40	
	Min/max number of iterations	100/500	

## Appendix B. Additional Results

- The tradeoff between cost and accuracy, a zoom-in view.

Figure A1 shows an enlargement of the graph that explores the tradeoffs between cost and accuracy (Figure 2 in the main text). It shows that 3–6 fold higher accuracy and cost were obtained for a sensor array deployed close to the site fence.

- The relationship between computed accuracy (*Ψ_Err_*) and the Euclidean discrepancy

Figure A2 shows the relationship between the computed *Ψ_Err_*, and the Euclidian discrepancy. Since the Euclidian discrepancy results from the problem settings (in the case of a simulation) or physical measurements (in a real case scenario) and *Ψ_Err_* is the optimization output, this correlation between these two measures is helpful for the design and allocation of the sensor array.

- The effect of MDL on the quantification and detection range

The effect of the MDL on method performance was explored for both a binary array and detectors with DR = 30. The top part of Figure A3 shows the performance of the detector array as a function of the MDL for a detector array with six detectors with a DR of 30. These results are compared to a six-binary sensor array (Figure A3 bottom).
